# Oxidative dehydrogenation of C–C and C–N bonds: A convenient approach to access diverse (dihydro)heteroaromatic compounds

**DOI:** 10.3762/bjoc.13.162

**Published:** 2017-08-15

**Authors:** Santanu Hati, Ulrike Holzgrabe, Subhabrata Sen

**Affiliations:** 1 Department of Chemistry, School of Natural Sciences, Shiv Nadar University, Dadri, Chithera, GautamBuddha Nagar, Uttar Pradesh 201314, India; 2 Institute of Pharmacy and Food Chemistry, University of Würzburg, Am Hubland, D-97074 Würzburg, Germany

**Keywords:** aerobic oxidation, bioinspired Flavin mimics, nitrogen heteroarenes, organo catalytic, oxidative dehydrogenation

## Abstract

Nitrogen heteroarenes form an important class of compounds which can be found in natural products, synthetic drugs, building blocks etc. Among the diverse strategies that were developed for the synthesis of nitrogen heterocycles, oxidative dehydrogenation is extremely effective. This review discusses various oxidative dehydrogenation strategies of C–C and C–N bonds to generate nitrogen heteroarenes from their corresponding heterocyclic substrates. The strategies are categorized under stoichiometric and catalytic usage of reagents that facilitate such transformations. The application of these strategies in the synthesis of nitrogen heteroarene natural products and synthetic drug intermediates are also discussed. We hope this review will arouse sufficient interest among the scientific community to further advance the application of oxidative dehydrogenation in the synthesis of nitrogen heteroarenes.

## Introduction

**Figure 1:**
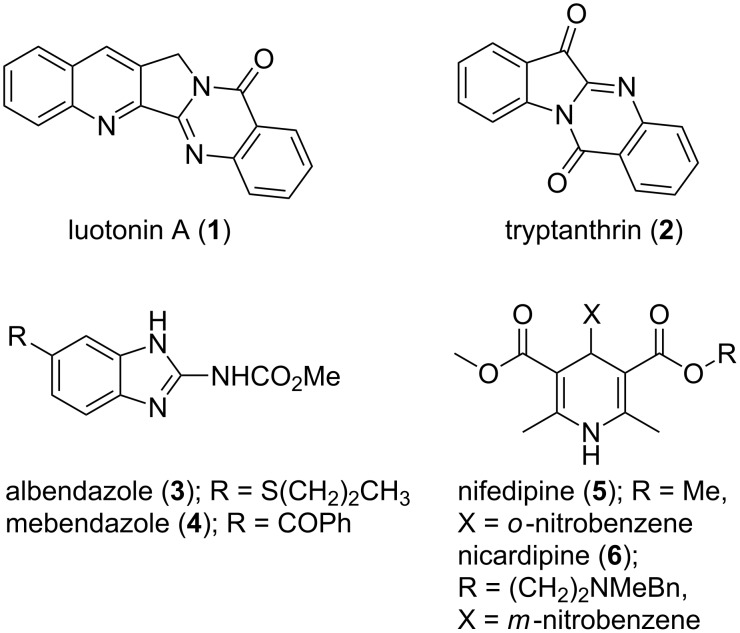
Representative bioactive heterocycles.

By virtue of their presence in bioactive natural products and active pharmaceutical ingredients, nitrogen heterocycles and heteroaromatics form an important class of compounds [1]. A large variety of such compounds are discovered so far and their therapeutic potential in diverse disease models has been thoroughly investigated [2]. For example thiazoles and oxazoles are found in various bioactive natural products, organic dyes and pharmaceutical intermediates [3–6]. Substituted benzimidazoles occur in veterinary medicines, as anthelmintic agents and are used in a plethora of human therapeutic areas such as psychiatrics, ulcers, hypertension, cancers etc. [7–10]. Quinazolines and quinazolones are obtained in bioactive alkaloids such as luotonin A, tryptanthrin and many more (Figure 1) [11–12]. Quinazoline derivatives work as potential inhibitors of epidermal growth factor (EGF) and tyrosine kinase receptors and also display antibacterial, antitubercular and antiviral properties [13–16]. Last but not the least, 1,4-dihydropyridines as calcium channel blockers, such as nifedipine and nicardipine, are found as effective chemotherapeutic medicines and antihypertensive agents [17].

Hence to attend their biological usefulness diverse synthetic processes are reported to access these molecules [18]. Multicomponent reactions are one of the key synthetic strategies that are applied to generate various nitrogen heterocycles and subsequent aromatization of these heterocycles afforded the corresponding heteroaromatic derivatives [19]. The aromatization of nitrogen heterocycles is facilitated mainly by dehydrogenation of the ring containing the nitrogen atom. The dehydrogenation strategy is an atom economical, efficient and sustainable method to access nitrogen containing heteroaromatic molecules. They can be either achieved by metals (like iridium, ruthenium, aluminum etc.) mediated processes (in absence of hydrogen acceptor) or via oxidative dehydrogenation in the presence of appropriate oxidants.

In absence of hydrogen acceptors, post dehydrogenation the hydrogen is released as H_2_↑. Catalysts such as iridium pincer complexes, CuAl_2_O_3_, hydroxyapatite bound palladium and ruthenium hydride complexes have been harnessed to facilitate such transformation [20–25].

**Scheme 1:**
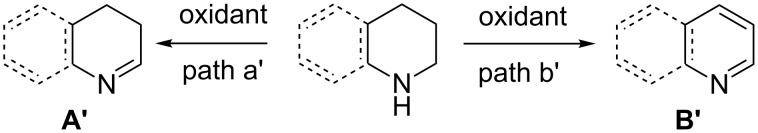
The concept of oxidative dehydrogenation.

In comparison, oxidative dehydrogenation of nitrogen heterocycles are mediated by an oxidant, which chelates with the nitrogen functionality of the heterocycles, and facilitates dehydrogenation by elimination (path a'). The dehydrogenation at times lead to complete aromatization of the moiety based on the dehydrogenation capability of the oxidant as well as the presence of the appropriate acidic protons in the heterocyclic substrate (path b', Scheme 1). To attend greener synthesis metal catalyzed and organocatalytic aerobic oxidative dehydrogenation strategies have been reported lately.

This review will discuss various techniques of oxidative dehydrogenation of nitrogen heterocycles via reagents, catalysts and also in the presence of stoichiometric oxidants or under aerobic conditions.

## Review

### Reagent-based approaches

For the reagent-based oxidative dehydrogenation, a stoichiometric amount of oxidant is applied in the reaction that gets associated with the nitrogen and facilitates subsequent proton abstraction and elimination in the ring to afford the desired heteroaromatics. Various organic oxidants such as 2-iodoxybenzoic acid (IBX), 2,3-dichloro-5,6-dicyano-1,4-benzoquinone (DDQ), KMnO_4_, transition metal-based oxidants and air have been extensively used to promote this transformation.

#### *o*-Iodoxybenzoic acid (IBX)-mediated oxidative dehydrogenation

**Scheme 2:**
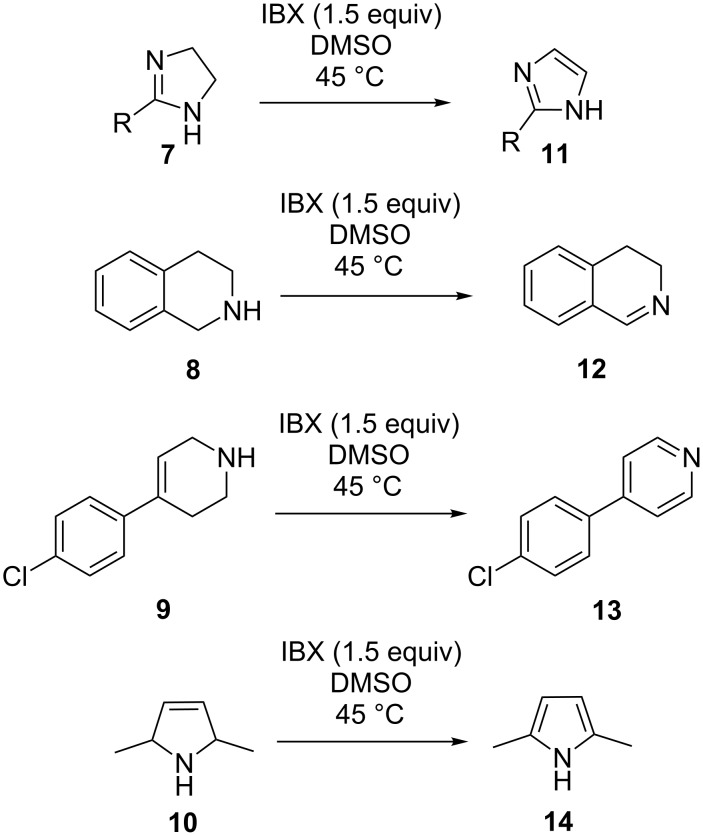
IBX-mediated oxidative dehydrogenation of various heterocycles [31–34].

IBX was first introduced as an oxidant (in oxidative dehydrogenation) by Nicolaou and co-workers in the year 2000 [26–29]. It oxidizes diverse functionalities such as amines, imines, alcohols etc. [30]. Later, it was demonstrated that IBX can also be used as a reagent for oxidative dehydrogenation of benzylic carbons in various aromatic systems via single electron transfer (SET) and/or ionic pathways [31–34]. It was not until 2004, that IBX was applied in the oxidative dehydrogenation of functionalized cyclic and acyclic ketones [31–34]. IBX was further harnessed in the oxidative dehydrogenation of 2°-amines, *N*-hydroxides, imines and oximes [31–34]. Encouraged by these results a variety of functionalized *N*-heterocycles were oxidatively dehydrogenated with IBX, to afford their aromatic counterpart. For example imidazoles **11**, dihydroisoquinoline **12**, pyridine **13**, and pyrrole **14** were obtained from their corresponding heterocyclic precursors **7**–**10** in excellent yields (Scheme 2) [31–34].

**Scheme 3:**
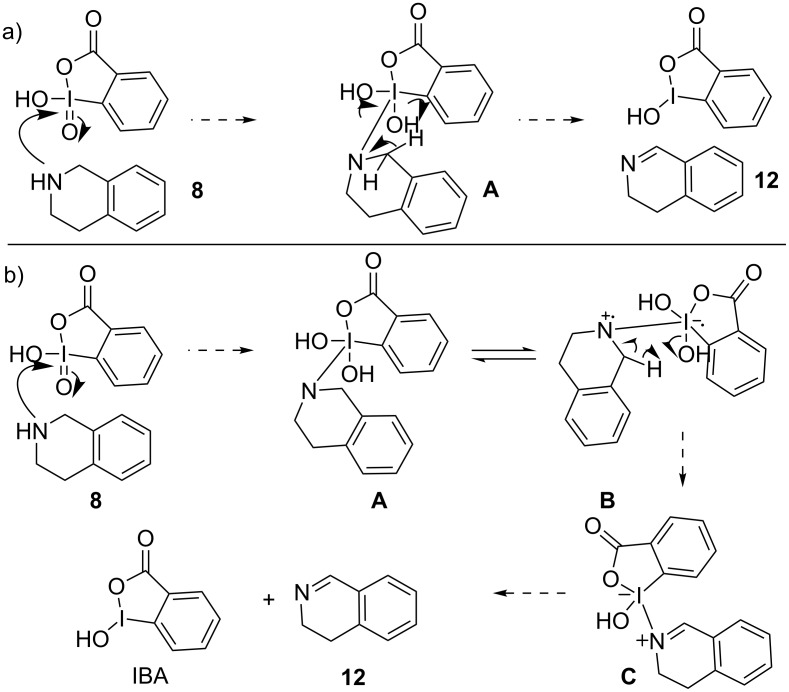
Potential mechanism of IBX-mediated oxidative dehydrogenation of *N*-heterocycles [31–34].

The general mechanistic pathway proposed for these transformations involved either an ionic or a SET mechanism. The emphasis was provided more on the ionic mechanism but a SET pathway cannot be excluded. The representative mechanism involved the coordination of the amine substrate with IBX, leading to the formation of **A** via reduction of the iodine center [31–34]. Intermediate **A** could then be converted to the desired heteroaromatic moiety **12**, either by elimination of water in a concerted fashion as proposed by the ionic pathway (Scheme 3a) or via a SET mechanism to generate a radical cation **B**, followed by fragmentation to afford **12** (Scheme 3b). Either of these processes provided the imine moiety, along with *o*-iodosobenzoic acid (IBA, Scheme 3).

**Scheme 4:**
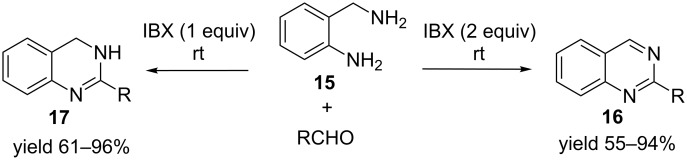
IBX-mediated room temperature one-pot condensation–oxidative dehydrogenation of *o*-aminobenzylamines.

In general, IBX-mediated oxidative dehydrogenation is conducted at high temperatures (>50 °C). Interestingly IBX explodes at ≈200 °C. Hence there is a hesitation to utilize IBX in the industry due to fear of explosion from “run-away” reactions. To alleviate this problem, IBX-mediated room temperature oxidative dehydrogenation can be utilized. Recently IBX-mediated oxidative dehydrogenation of tetrahydroquinazolines has been demonstrated at room temperature. The reaction involved a one-pot condensation–oxidative dehydrogenation of 2-aminobenzylamine (**15**) with appropriate aldehydes to dihydroquinazoline **17** and aromatic quinazolines **16** with 1 or 2 equivalents of IBX, respectively (Scheme 4) [35]. The mechanism of conversion could be similar to the one depicted in Scheme 3.

**Scheme 5:**
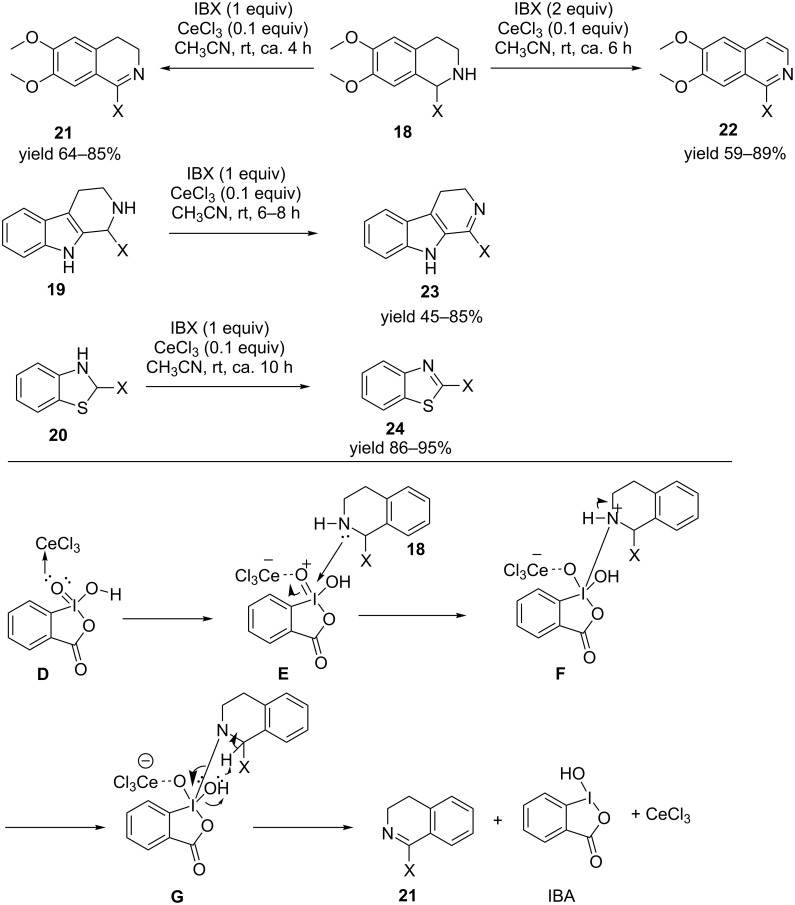
Anhydrous cerium chloride-catalyzed, IBX-mediated oxidative dehydrogenation of various heterocycles at room temperature.

In another example anhydrous cerium chloride was used as a co-catalyst to activate IBX towards oxidative dehydrogenation of various heterocycles such as 1-substituted tetrahydroisoquinolines **18**, tetrahydro-β-carbolines **19** and benzothiazolidines **20** to their corresponding heterocyclic analogs **21**–**24** at room temperature [36]. Anhydrous cerium chloride is believed to coordinate with IBX to increase the electrophilicity of the iodine center and thereby generating **F**, where the coordination of the amine substrate becomes much more facile thereby promoting the reaction at room temperature (Scheme 5).

#### Transition metal-free approaches for oxidative dehydrogenation

**Scheme 6:**
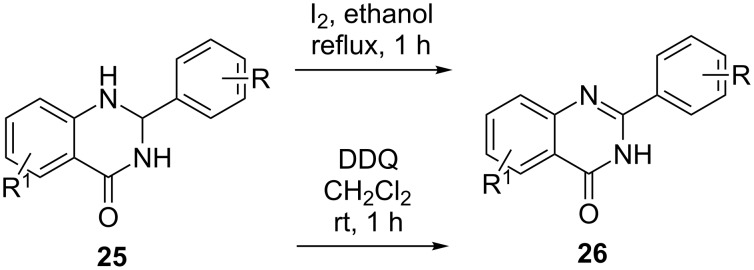
Oxidative dehydrogenation of quinazolinones with I_2_ and DDQ [37–40].

Apart from IBX, various other oxidants (non-metallic) have been applied for oxidative dehydrogenation of C–C and C–N bonds. The synthesis of heteroaromatic compounds such as quinazolinones were facilitated by iodine and DDQ-mediated oxidative dehydrogenation as depicted in Scheme 6 [37–40]. Typically, quinazolinone **25** was refluxed in the polar solvent ethanol with iodine to afford the dihydro derivative **26** (Scheme 6). Interestingly, DDQ facilitated similar reactions at room temperature.

**Scheme 7:**
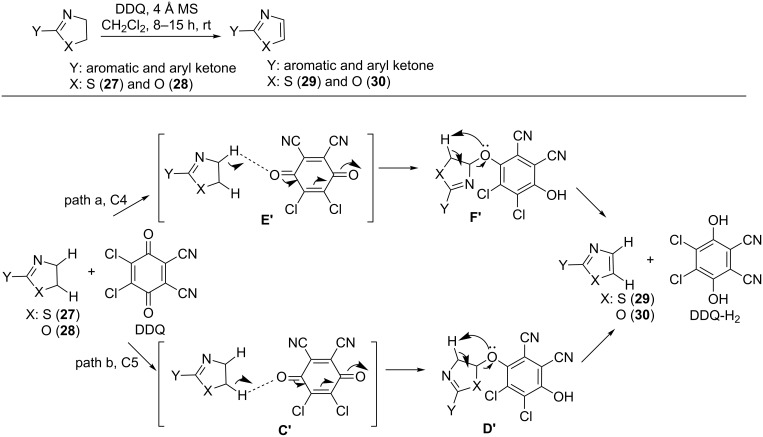
DDQ-mediated oxidative dehydrogenation of thiazolidines and oxazolidines.

DDQ also induced oxidative dehydrogenation in 2-thiazolidines **27** and 2-oxazolidines **28** at room temperature in the presence of 4 Å molecular sieves with dichloromethane as solvent to generate diversely substituted 2-thiazoles **29** and 2-oxazoles **30** (Scheme 7) [41]. The putative mechanism initiated with the reaction of DDQ on the C4 H-atom of **27** and **28**, respectively, to form the transition state **E′** (path a). Alternatively it can also attack the H-atom at C5 to generate **C′** (path b). A single electron transfer and subsequent H-abstraction on **E′** or **C′** lead to the formation of **F′** or **D′**, respectively. **F′** or **D′** undergoes H-migration to generate **29** and **30**, respectively, along with DDQ-H_2_. A further investigation revealed that path b is the preferred pathway for this transformation (Scheme 7) [41].

**Scheme 8:**
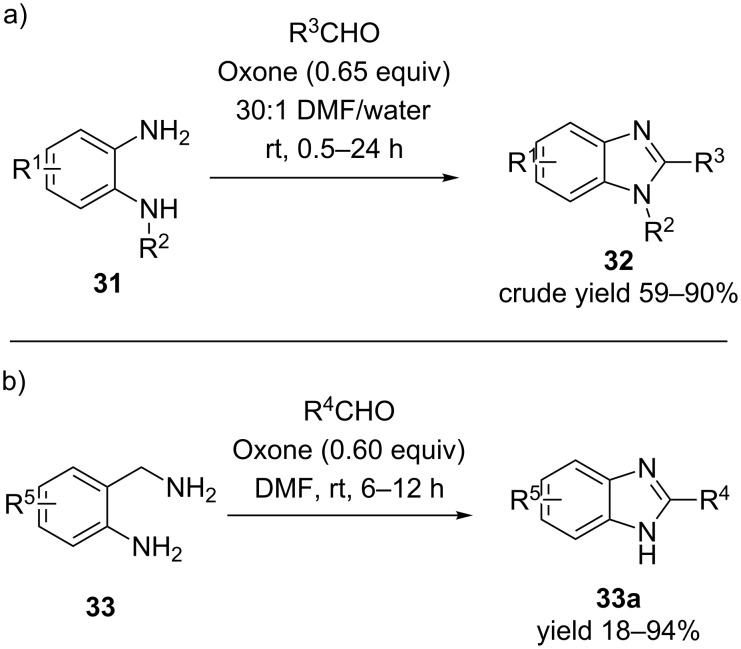
Oxone-mediated oxidative dehydrogenation of intermediates from *o*-phenylenediamine and *o*-aminobenzylamine [42–43].

An economical and simple procedure for the oxidative dehydrogenation is demonstrated by the usage of ≈0.6 equivalents of oxone as an appropriate oxidant in wet dimethylformamide, at room temperature for the reaction of *o*-phenylenediamine **31** with appropriate aldehydes to afford 2-substituted benzimidazoles **32** in excellent yield [42]. The reaction conditions are amenable to a wide range of substrates including aliphatic, aromatic and heteroaromatic aldehydes. Neither the steric nor the electronic properties of the substituents on the aldehydes affect the yield of the reaction. In general, the crude products were isolated by simple extraction or precipitation from the reaction mixture in moderate to excellent yield. The only limitation observed in this procedure is the use of aldehydes that contain functionalities which are susceptible to oxidation in the presence of oxone, failed to generate the desired products (Scheme 8a).

An interesting application of oxone in the synthesis of 2-substituted benzimidazoles **33a**, involved a one-pot condensation–ring distortion–oxidative dehydrogenation of *o*-aminobenzylamines **33** and appropriate aldehydes [43]. The reaction conditions included 0.6 equiv of oxone at room temperature with DMF as the solvent. Various aliphatic, aromatic and heteroaromatic aldehydes are reacted with *o*-aminobenzylamine and substituted *o*-aminobenzylamines to provide the desired products in decent yields (Scheme 8b).

**Scheme 9:**
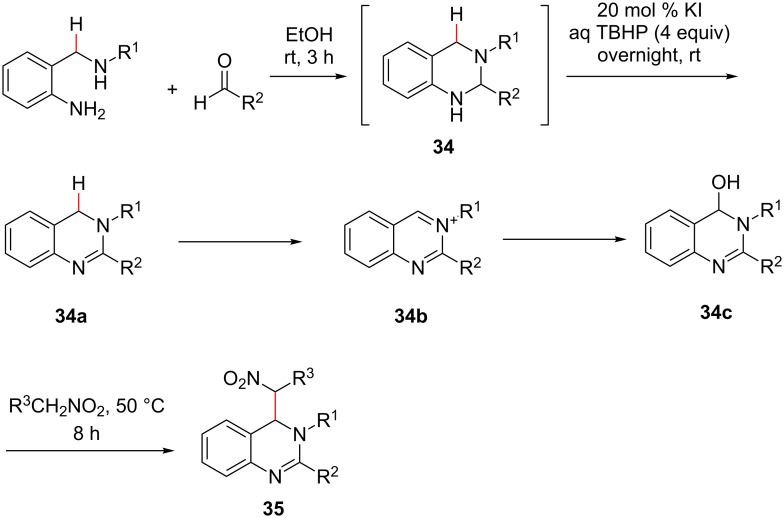
Transition metal-free oxidative cross-dehydrogenative coupling.

Kumar et al. demonstrated transition metal-free α-C(sp^3^)–H bond functionalization of amines via an oxidative cross-dehydrogenative coupling reaction [44]. They reported a one-pot synthesis of substituted dihydroquinazolines **35** from a variety of *o*-aminobenzylamines with appropriate aldehydes and alkylnitrates. The initial condensation of substituted *o*-aminobenzylamines with aldehydes afforded the substituted tetrahydroquinazoline **34**, which was subsequently treated with a catalytic amount of potassium iodide (0.2 mmol) and 0.25 mL of *tert*-butylhydroperoxide (TBHP, 70 wt %) in water (4 equiv) to afford the dihydroisoquinazoline **34a**, which got oxidized to the quinazolium intermediate **34b**. Hydroxylation of **34b** afforded **34c**, which was further reacted with nitroalkanes at 50 °C to generate the desired substituted dihydroquinazolines **35** in moderate to excellent yields (Scheme 9) [44].

**Scheme 10:**
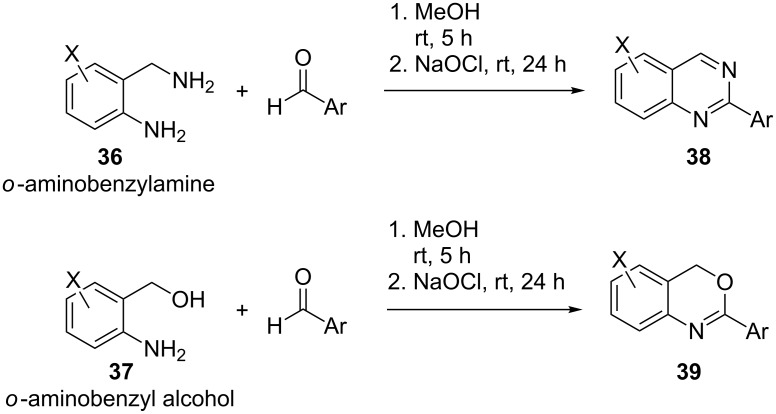
NaOCl-mediated oxidative dehydrogenation.

In another example commercially available sodium hypochlorite (NaOCl) was used as an oxidant, under mild conditions in a highly efficient oxidative dehydrogenative coupling of *o*-aminobenzylamines and *o*-aminobenzyl alcohols **36** and **37**, respectively, with appropriate aldehydes to generate substituted quinazolines **38** and 4*H*-benzo[*d*][1,3]oxazines **39** in excellent yields [45]. A typical procedure involved the condensation of **36** and **37** in methanol at room temperature to afford the crude heterocycles which were then further treated with NaOCl (≈3.0 equiv) in the same pot at room temperature to afford the desired products which were then purified by column chromatography (Scheme 10).

**Scheme 11:**
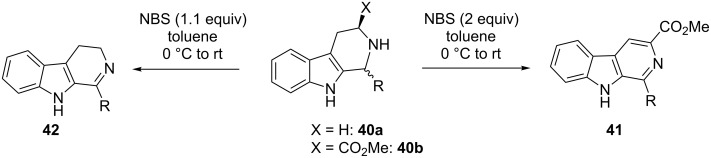
NBS-mediated oxidative dehydrogenation of tetrahydro-β-carbolines.

Recently, a facile *N*-bromosuccinimide (NBS) induced oxidative dehydrogenation of diversely substituted tetrahydro-β-carbolines **40a**,**b** were reported to generate aromatic β-carbolines **41** and 3,4-dihydro-β-carbolines **42** in moderate to good yields [46]. Typically the reaction occurs in toluene at 0 ºC to room temperature and is completed within 6 h. When **40a** was treated with 1.1 equivalents of NBS it furnished **42** and when **40b** was reacted with 2 equivalents of the same reagent, it afforded **41**. The ester functionality on **40b** facilitated the complete aromatization of the ring to afford the desired products (Scheme 11).

**Scheme 12:**
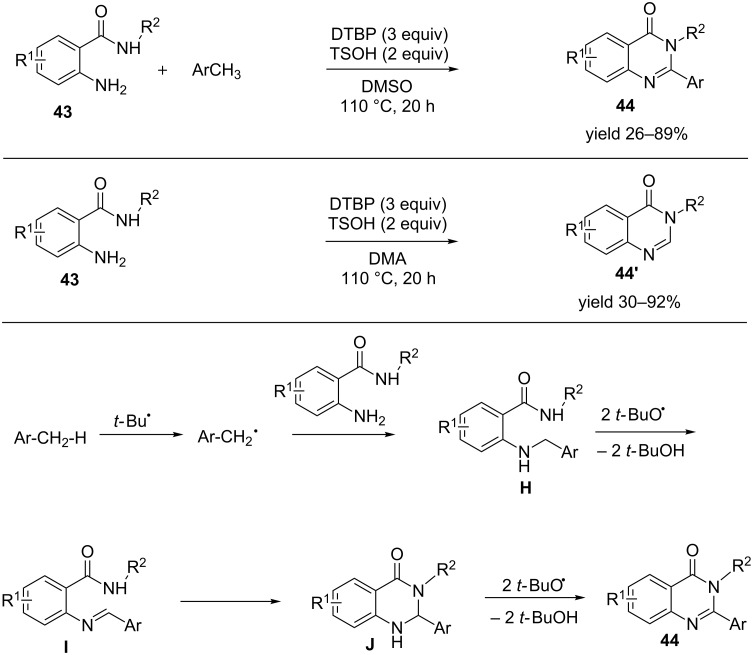
One-pot synthesis of various methyl(hetero)arenes from *o*-aminobenzamide in presence of di-*tert*-butyl peroxide (DTBP).

It is always a tedious effort to activate a sp^3^ C–H bond towards any transformation. A method that enables such transformation requires special mention. Herein we indicate such an elegant report where *o*-aminobenzamide **43** were reacted with various methyl(hetero)arenes in the presence of di-*tert*-butyl peroxide (DTBP, 0.9 mmol), *p*-toluenesulfonic acid (0.6 mmol) in DMSO at 110 °C for ≈20 hours to facilitate a variety of quinazolinones **44** (Scheme 12) [47]. *N*-Alkyl benzamides **44´** were also synthesized with this protocol. The average yield ranged from 30 to 92%. Other than methyl(hetero)arenes dimethylamides were also used as C-synthon for such intermolecular annulation to afford unsubstituted quinazolinones. A putative mechanism involved the homolysis of DTBP to generate *tert*-butoxide radicals, which in turn abstracts a proton radical from the methyl(hetero)arene to facilitate the formation of a benzylic radical. The benzylic radical couples with *o*-aminobenzylamide to generate **H**. It then interacts with the *tert*-butoxide radical to generate **I**. **I** then undergoes an intramolecular cyclization to form **J**, followed by aromatization to afford the desired compounds.

#### Transition metal-induced oxidative dehydrogenation

Since the applications of various transition metals as catalysts in C–N, C–O and C–C bond forming reactions, they have become very popular reagents in synthetic organic chemistry. Their utility has been explored in various organic reactions including oxidative dehydrogenation of heterocycles. A few of such metal-mediated reactions are discussed below.

**Scheme 13:**
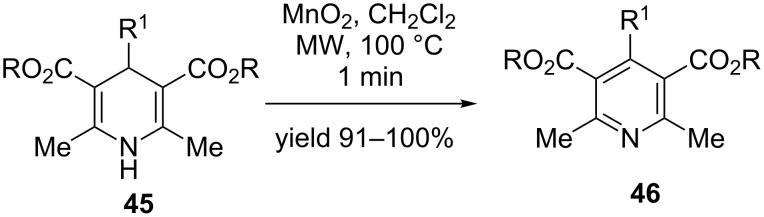
Oxidative dehydrogenation of 1, 4-DHPs.

By virtue of its immense pharmaceutical significance Hantzsch 1,4-dihydropyridines (1,4-DHP) have garnered substantial attention in the scientific community [48]. They are found in various chemotherapeutic agents and are used for the treatment of cardiovascular diseases such as hypertension and angina pectoris [49]. They are an important class of calcium channel blockers that reduces the transmembrane calcium current upon binding, thereby relaxing the heart muscles [50]. Interestingly, 1,4-DHP drugs are metabolized in the liver by CYP-450 enzymes and undergo oxidative dehydrogenation to generate the corresponding pyridine derivatives [51]. To understand and model these biological pathways, oxidative aromatization of 1,4-DHP to their corresponding pyridine derivatives has acclaimed wide attention. A variety of oxidants such as urea nitrate, BrCCl_3_/hν, nitric acid, nitric oxide, *N*-methyl-*N*-nitroso-*p*-toluenesulfonamide, DDQ etc. has been used to facilitate the conversion [52–55]. Unfortunately most of these methods suffer from the disadvantages of prolonged reaction time, poor yield and competing oxidative dealkylation of 4-benzyl and *sec*-alkyl-substituted DHP substrates. Alternative strategies involving transition metals and microwave heating, ultrasonication and solvent-free conditions alleviated the drawbacks to a major extent [56–61]. One such useful strategy include manganese dioxide (MnO_2_)-mediated oxidative aromatization of 1,4-DHP **45** to afford substituted pyridine derivatives **46** under microwave conditions (Scheme 13) [62]. This approach drastically reduces the reaction time to ≈1 minute and provided the desired products in excellent yields (Scheme 13).

**Scheme 14:**
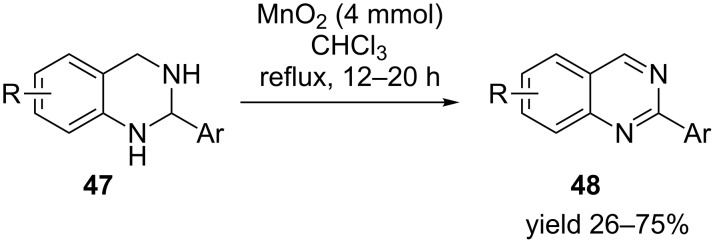
Synthesis of quinazolines in the presence of MnO_2_.

In another example Peng et al. have demonstrated the use of manganese oxide (MnO_2_) as an oxidant in the synthesis of 2-arylquinazolines from its heterocyclic precursors [63]. A variety of substituents were well tolerated on both quinazoline and on the appended aromatic ring to generate the desired compounds **48** in moderate to excellent yield. The reaction conditions involved refluxing of 2-aryl-1,2,3,4-tetrahydroquinazoline **47** in chloroform for twelve to twenty hours (Scheme 14). In a similar effort, quinazolinones were synthesized by potassium permanganate-mediated oxidative dehydrogenation in the presence of acetone under reflux conditions [64].

**Scheme 15:**
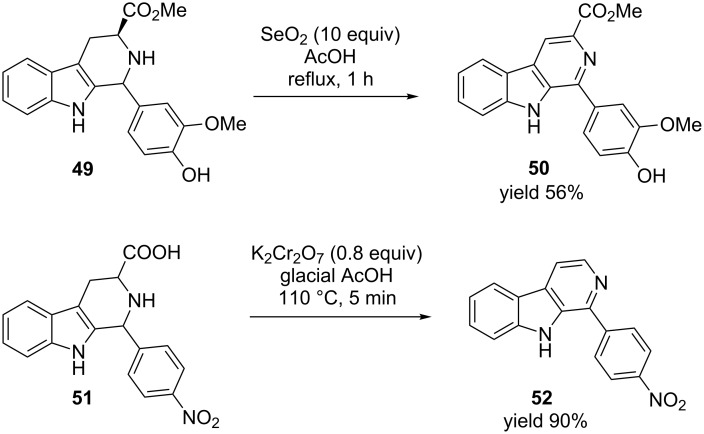
Selenium dioxide and potassium dichromate-mediated oxidative dehydrogenation of tetrahydro-β-carbolines [65–66].

Various aromatic β-carbolines has been accessed by oxidative dehydrogenation of their heterocyclic precursors using metal-based reagents. For example selenium dioxide (SeO_2_, 10 equiv) has been used in the presence of an acid for oxidative dehydrogenation of tetrahydro-β-carbolines **49** to afford aromatic β-carbolines **50** (Scheme 15) [65]. Bhutania et al. [66] has also reported a similar synthesis of 1-aryl-β-carboline derivative **52** starting from **51** (Scheme 15). In this case potassium dichromate (K_2_Cr_2_O_7_) was used as an oxidant in glacial acetic acid. The reaction completes within 5 minutes with excellent yield of the final product.

**Scheme 16:**
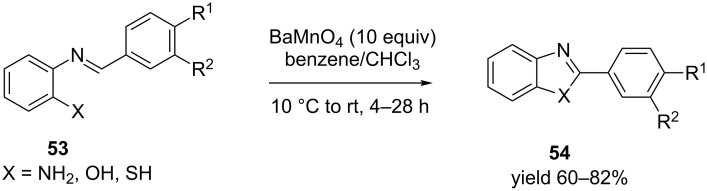
Synthesis of substituted benzazoles in the presence of barium permanganate.

Not very many metal oxidants have been explored to transform aromatic Schiff's bases to heterocycles. In one such elegant effort, Venkataramani et al. [67] used barium manganate as an oxidant. The initial condensation of different *o*-phenylenediamine, *o*-aminophenol or *o*-aminothiophenol with aromatic aldehydes produced the corresponding Schiff's bases **53** which underwent a one-pot cyclisation and oxidative dehydrogenation in the presence of barium manganate to produce benzimidazoles, benzoxazoles and benzothiazoles **54**, respectively in appropriate solvent at ambient or lower temperature (Scheme 16).

### Catalytic approaches

As a normal evolution of any synthetic approaches, oxidative dehydrogenation also progressed from reagent-based approaches to catalytic methodologies. Aerobic oxidation in the presence of bioinspired and metal catalysts as well as synthetic oxidants-based metal-catalyzed reactions bolstered the application of this strategy in accessing various heteroarenes. In this section we discuss a few of such examples.

#### Bioinspired aerobic dehydrogenation

**Scheme 17:**
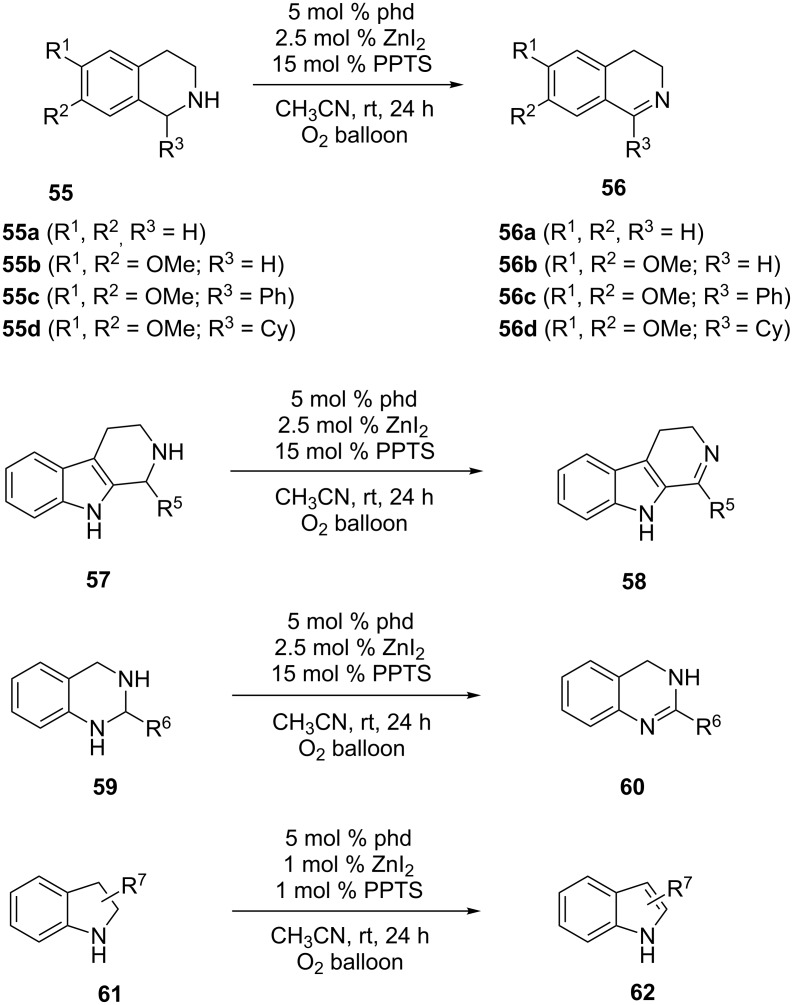
Oxidative dehydrogenation with phenanthroline-based catalysts. PPTS = pyridinium *p*-toluenesulfonic acid, phd = 1,10-phenanthroline-5,6-dione.

The bioinspired aerobic dehydrogenation strategy is an environmentally “green” way to access heteroaromatic moieties from their heterocyclic precursors [68]. The approach is inspired from enzyme catalyzed reactions where organic cofactors are used as naturally occurring oxidases and oxygenases [69]. One of the early examples of bioinspired aerobic oxidation involved copper amine oxidation to facilitate the transformation of amines to aldehydes [70–71]. Recently, several examples of quinine-based catalysts as alternatives to metal-based catalysts are reported, that facilitates oxidative dehydrogenation of amines [72–80]. An exquisite example in this aspect involved phenanthroline-based catalysts that are applied for oxidative dehydrogenation of 2°-amines [81]. In this regard, Stahl and co-workers showed that coordination of 1,10-phenanthroline-5,6-dione (phd) with Zn^2+^ salts enhances the catalytic capacity of phd towards oxidative dehydrogenation of a variety of nitrogen heterocycles (Scheme 17). Thorough investigation and isolation of Zn-phd complexes revealed that the coordination of Zn^2+^ with the remote nitrogen of phd rationalizes the efficiency of this system towards oxidative dehydrogenation of 2°-amines. To assess the substrate scope, various secondary amines were successfully oxidized in the presence of catalytic Zn-phd complexes to provide the corresponding oxidized product. The optimized catalytic system facilitated the conversion of tetrahydroisoquinolines **55a** to 3,4-dihydroisoquinolines **56a** (Scheme 17). Electron-donating groups improved the reaction yield with reduced reaction time. Various analogs **56b**–**d** with both aryl and alkyl substitution at C1 were synthesized. In another example tetrahydro-β-carbolines **57** were readily converted to 3,4-dihydro-β-carbolines **58** under the same optimized conditions. Aryl substitution at C1 was well tolerated. Quinazolines **60** were formed in an efficient manner from tetrahydroquinazolines **59**. Unlike other heterocycles, the reactions with electron-withdrawing substrates were favored in this case. A slight modification (5 mol % phd, 1 mol % ZnI_2_ and 1 mol % PPTS) of the existing procedure afforded indoles **62** from indolines **61**.

**Scheme 18:**
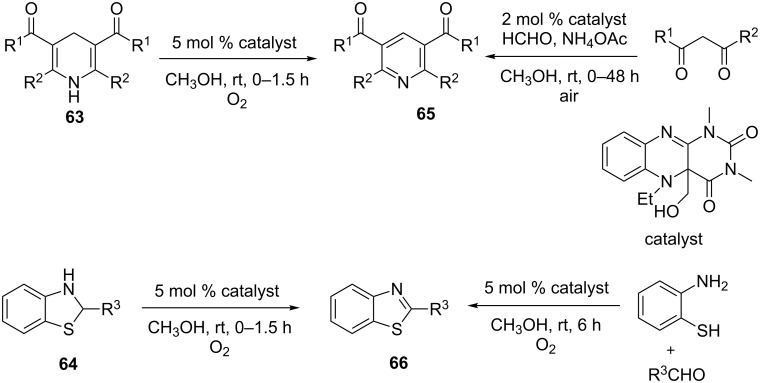
Oxidative dehydrogenation with Flavin mimics.

Bioinspired flavin mimics were also used as catalysts for oxidative dehydrogenation of dihydropyridines **63** and benzothiazolines **64** to substituted pyridines **65** and benzothiazoles **66**, respectively, in moderate to excellent yields (Scheme 18) [82]. The reaction was performed in methanol at ambient temperature. The optimized procedure also generated **65** and **66** from a one-pot reaction of various dicarbonyls, formaldehyde, ammonium acetates, *o*-aminothiophenols and various aldehydes, respectively (Scheme 18).

**Scheme 19:**
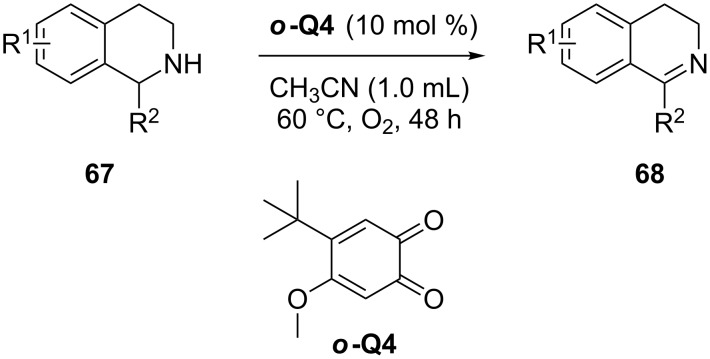
*o*-Quinone based bioinspired catalysts for the synthesis of dihydroisoquinolines.

In another example an *ortho*-quinone-based bioinspired catalyst was used in the oxidative dehydrogenation of tetrahydroisoquinolines **67** to dihydroisoquinoline **68** in acetonitrile at 60 °C under oxygen atmosphere (Scheme 19) [83]. In general, electron-donating and neutral functionality (R = OMe, H) on the tetrahydroisoquinolines were well tolerated. Interestingly the electron-withdrawing groups were conspicuous by their absence.

#### Metal-catalyzed aerobic dehydrogenation

**Scheme 20:**
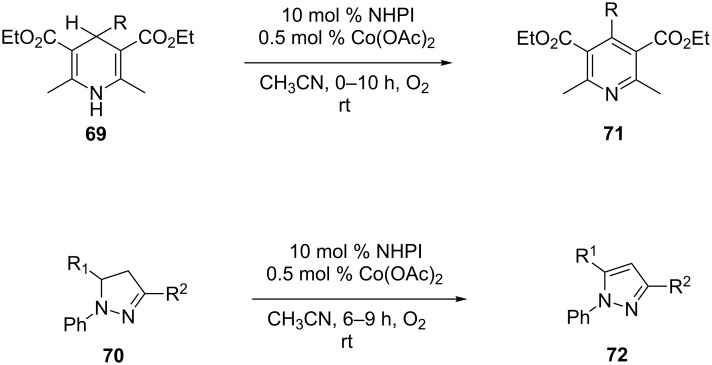
Cobalt-catalyzed aerobic dehydrogenation of Hantzch 1,4-DHPs and pyrazolines.

From organocatalytic aerobic dehydrogenation, in this section we turn our attention to transition metal-catalyzed aerobic dehydrogenation of C–C and C–N bonds. Molecular oxygen is well established as an oxidant for oxidative dehydrogenation of heterocycles. These reactions occur under open air or excess oxygen pressure. They are classified as aerobic oxidation. One of the early examples of such reaction was demonstrated by Han et al. in 2006 [84]. Here in pyridines **71** and pyrazoles **72** were synthesized in excellent yield by the oxidation of 4-substituted Hantzsch 1,4-dihydropyridines **69** and 1,3,5-trisubstituted pyrazolines **70** via molecular oxygen. The reaction was facilitated at room temperature by *N*-hydroxyphthalimide (NHPI) and cobalt acetate (Co(OAc)_2_) as catalysts in acetonitrile (Scheme 20).

**Scheme 21:**
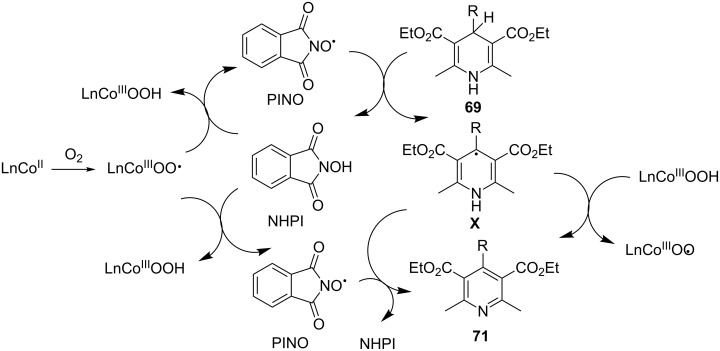
Mechanism of cobalt-catalyzed aerobic dehydrogenation of Hantzch 1,4-DHPs.

The reaction followed a free radical mechanism as exemplified by the oxidative dehydrogenation of DHPs. The initial step involved the formation of the phthalimide-*N*-oxyl radical (PINO) via transfer of hydrogen from NHPI to O_2_. Co^2+^-assisted this step by associating with oxygen to generate a Co^3+^–oxygen complex. It then abstracts the hydrogen from NHPI. Next, PINO abstracted a hydrogen from the DHP produced to generate radical **X** which aromatizes via hydrogen abstraction by PINO and/or the Co^3+^–oxygen complex to provide the pyridine derivatives (Scheme 21).

**Scheme 22:**
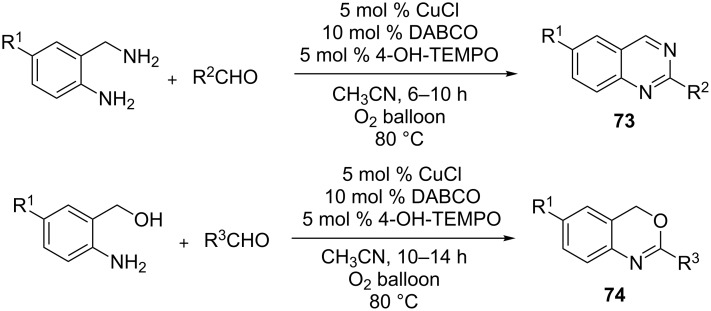
DABCO and TEMPO-catalyzed aerobic oxidative dehydrogenation of quinazolines and 4*H*-3,1-benzoxazines.

One of the other noteworthy examples is a copper chloride/1,4-diazabicyclo[2.2.2]octane (DABCO) and 4-hydroxy-2,2,6,6-tetramethyl-1-piperidinyloxy (TEMPO)-catalyzed aerobic oxidative dehydrogenation approach for the synthesis of quinazolines **73** and 4*H*-3,1-benzoxazines **74** [85]. This is the first time that such a catalytic system was harnessed for successful oxidation of heterocycles (a similar transformation was achieved with NaOCl as the oxidant (Scheme 10)). It demonstrates the successful generation of the aforementioned heteroaromatic compounds in a one-pot reaction from aldehydes with 2-aminobenzylamines and 2-aminobenzyl alcohols with molecular oxygen as the oxidant (Scheme 22). It is a simple and environmentally benign protocol for the generation of these heterocycles (Scheme 22) in excellent yield.

**Scheme 23:**
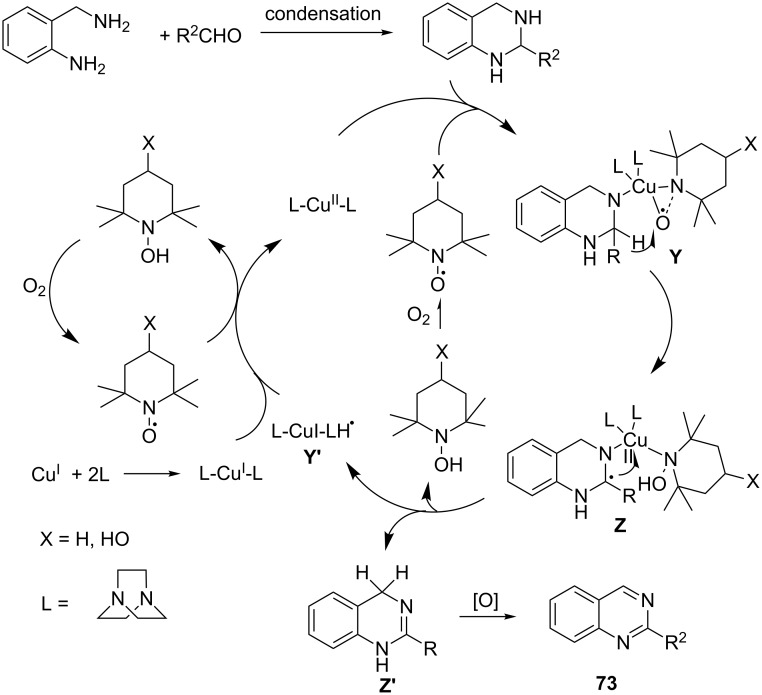
Putative mechanism for Cu(I)–DABCO–TEMPO catalyzed aerobic oxidative dehydrogenation of tetrahydroquinazolines.

The proposed mechanism of this transformation is illustrated in the formation of **73**. It involved oxidation of Cu(I)-(DABCO)_2_ by either oxygen or TEMPO to afford the Cu(II)-(DABCO)_2_ complex which gets coordinated with the *N*-atom of the substrate and TEMPO to generate an η_2_ complex **Y**. The benzylic hydrogen is then transferred to TEMPO resulting in a radical–TEMPO–Cu intermediate **Z**. The benzyl radical in **Z** is then further oxidized to the corresponding carbocation which gets deprotonated to afford dihydroquinazoline **Z´** along with **Y´** and TEMPOH. Finally, **Z´** is further oxidized to **73**. TEMPOH oxidizes back to TEMPO which further repeats the catalytic cycle (Scheme 23).

**Scheme 24:**
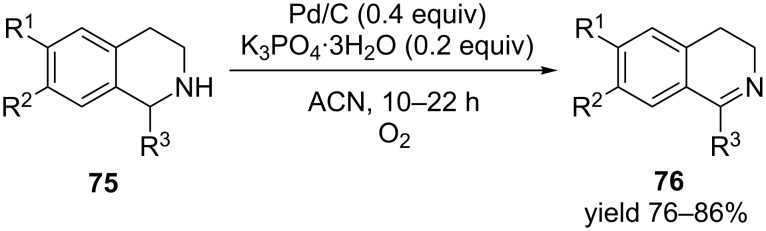
Potassium triphosphate modified Pd/C catalysts for the oxidative dehydrogenation of tetrahydroisoquinolines.

In another interesting example 1-substituted-1,2,3,4-tetrahydroisoquinolines were selectively dehydrogenated in the presence of catalytic Pd/C, modified by potassium phosphate trihydrate (K_3_PO_4_·3H_2_O) under oxygen atmosphere [86]. Original Pd/C lead to sluggish reactions compared to the modified form. The catalyst could also be recycled at least thrice (Scheme 24). This facile synthesis demonstrated seamless production of dihydroisoquinolines. They were the predominant products with nearly 3–5% formation of the aromatic isoquinoline (Scheme 24).

**Scheme 25:**
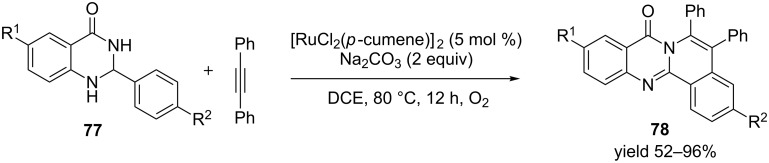
Ruthenium-catalyzed polycyclic heteroarenes.

Ruthenium catalysts have also been used under aerobic conditions for oxidative dehydrogenation of heterocycles. For example Lingayya et al. demonstrated that ruthenium chloride (*p*-cumene)_2_ [RuCl_2_(*p*-cumene)_2_] catalyzed tandem reaction involving oxidative dehydrogenation, cross coupling and annulation of dihydroquinazolinones **77** with diphenylacetylene to generate polycyclic heteroarenes **78** under oxygen atmosphere (Scheme 25) [87]. Diversely substituted quinazolinones were reacted with diphenylacetylene to generate the target compounds in excellent yields.

**Scheme 26:**
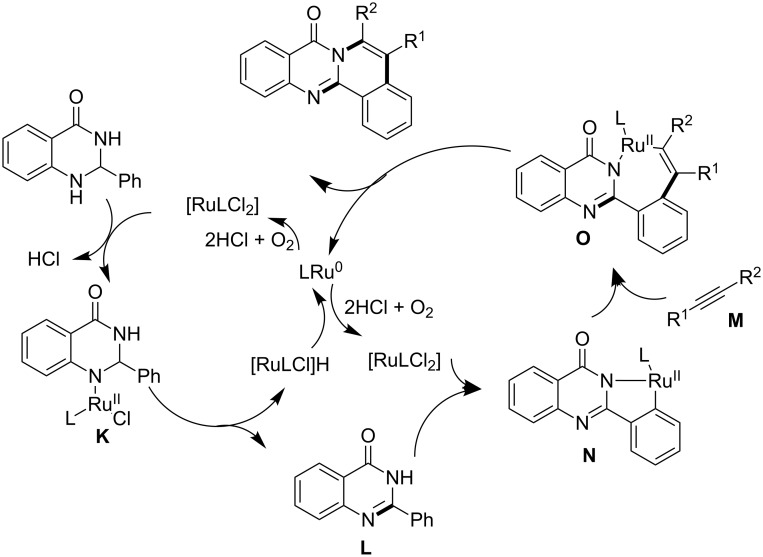
Plausible mechanism of the ruthenium-catalyzed dehydrogenation.

A plausible mechanistic rational involved the coordination of L_2_RuCl_2_ (L = *p*-cumene) with the more basic quinazolinone nitrogen to form complex **K**, which underwent β-H elimination to afford quinazolinone **L** and (RuLCl)-H, which further reduced to Ru^0^. Oxygen revived active Ru^I^ from Ru^0^. The neat step involved cross coupling/annulation of **L** with alkyne **M**. This is facilitated by coordination of **L** with Ru^II^, followed by C–H activation to afford **N**. Migratory insertion of **M** on **N** generate **O** and subsequent reductive elimination of **O** afforded the desired compounds (Scheme 26).

**Scheme 27:**
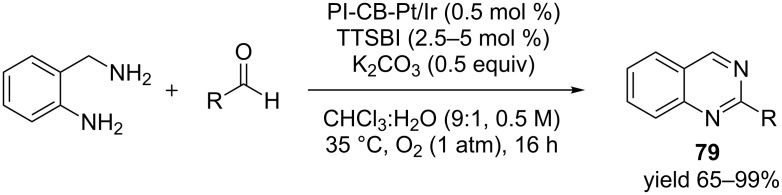
Bi-metallic platinum/iridium alloyed nanoclusters and 5,5’,6,6’-tetrahydroxy-3,3,3’,3’-tetramethyl-1,1’-spiro-bisindane (TTSBI) for the synthesis of quinazolines.

In another example Yuan et al. [88] demonstrated that a catalytic combination of heterogeneous polymer-supported bi-metallic platinum/iridium alloyed nanoclusters and 5,5’,6,6’-tetrahydroxy-3,3,3’,3’-tetramethyl-1,1’-spiro-bisindane (TTSBI) facilitated a one-pot condensation and oxidative dehydrogenation of *o*-aminobenzylamine to generate quinazoline derivatives **79** under mild aerobic conditions (Scheme 27). Low catalyst loading was required with a diverse substrate scope. The robustness of the reaction was demonstrated via gram-scale synthesis of the desired product.

**Scheme 28:**
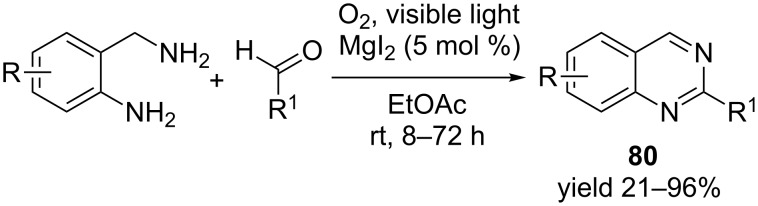
Magnesium iodide-catalyzed synthesis of quinazolines.

Recently, an aerobic photooxidative synthesis of quinazoline from 2-aminobenzylamine and aldehyde in the presence of visible light and catalytic MgI_2_ was described [89]. The reaction takes about 8–72 hours to complete depending on the substituents present in starting amines and aldehydes (Scheme 28). Initial condensation of 2-aminobenzylamine with an aldehyde generates 2-aryl-1,2,3,4-tetrahydroquinazolines which underwent oxidative dehydrogenation in the presence of magnesium iodide (that generates iodine) to afford the aromatic quinazoline **80** in excellent yield.

**Scheme 29:**
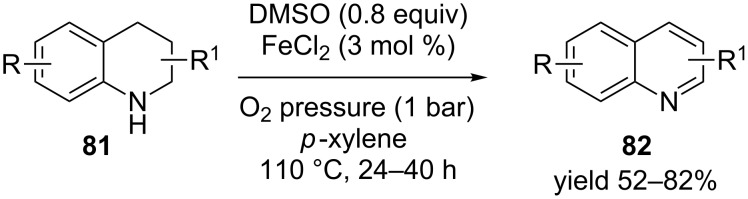
Ferrous chloride-catalyzed aerobic dehydrogenation of 1,2,3,4-tetrahydroquinolines.

Zhou et al. [90] reported a similar example in the presence of catalytic ferrous chloride and molecular oxygen for the synthesis of isoquinoline **82** from diverse 1,2,3,4-tetrahydroquinolines **81** in moderate to good yield (Scheme 29). Ferrous chloride (FeCl_2_) acted as a single electron transfer agent in the presence of DMSO to facilitate the reaction.

#### Metal-catalyzed oxidant induced dehydrogenation

**Scheme 30:**
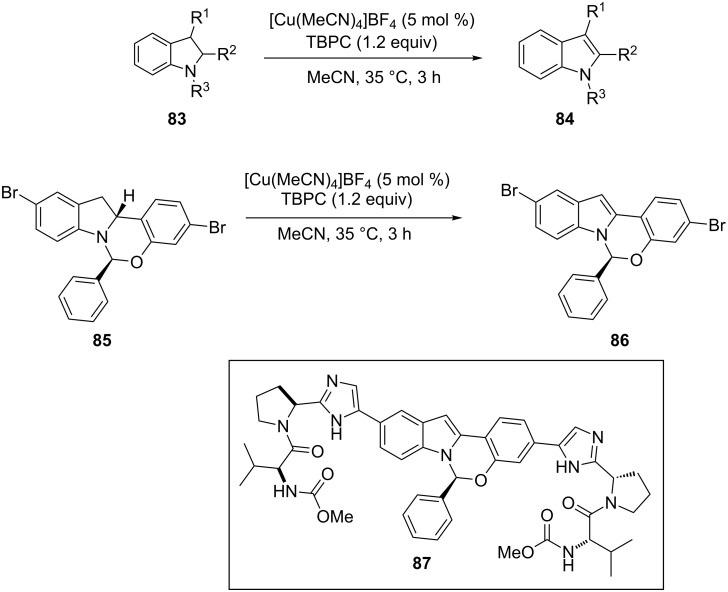
Cu(I)-catalyzed oxidative aromatization of indoles.

Our next discussion involved the oxidative dehydrogenation of C–C and C–N bonds utilizing metal catalysts with synthetic oxidants. The first example is illustrated by transforming an indoline to an indole [91]. In general, indoles are one of the most popular moieties that are observed in a plethora of bioactive natural products and active pharmaceutical ingredients. Peng et al. reported a novel catalyst oxidant combo of ([Cu(MeCN)_4_]BF_4_) and *tert*-butylperoxy 2-ethylhexyl carbonate (TBPC) to generate the corresponding indoles **84** in excellent yields from indolines **83** (Scheme 30) [91]. The methodology was further utilized in the conversion of benzoxazinoindolines **85** to benzoxazinoindoles **86** (found in elbasvir (**87**), a component of zepatier^®^, a combination therapeutic drug approved by FDA for curing Hepatitis C, Scheme 30). The robustness of the strategy is exemplified by demonstrating a successful scale-up reaction of **86** in 0.5 kg scale.

**Scheme 31:**
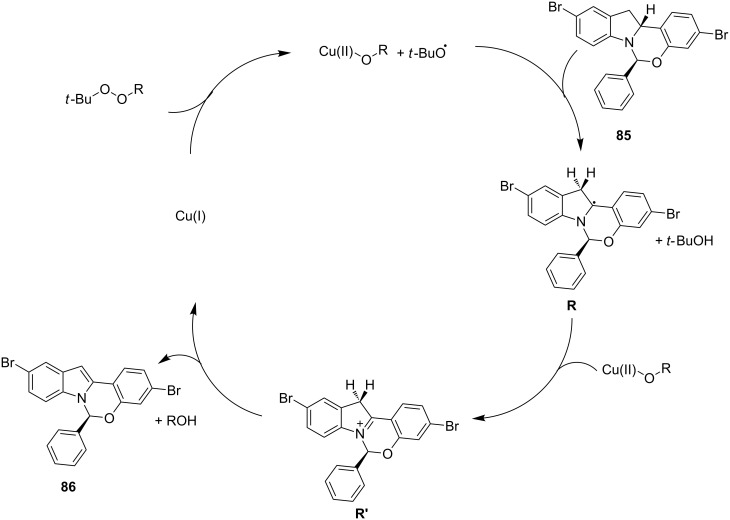
Putative mechanism of the transformation.

The putative mechanism of this transformation involved a single electron transfer reaction of the Cu(I) catalyst with TBPC to afford a *tert*-butoxy radical, which then reacts with **85** by abstracting a hydrogen atom to afford carbon radical species **R**. Cu(II)-mediated Kochi-type radical oxidation of **R** generated the iminium intermediate **R´** which undergoes aromatization to provide the desired **86** (Scheme 31).

**Scheme 32:**
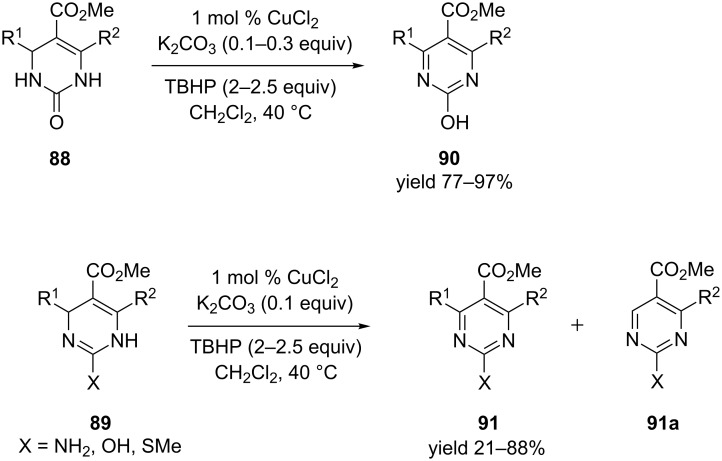
Oxidative dehydrogenation of pyrimidinones and pyrimidines.

Yamamoto et al. demonstrated a mild oxidative dehydrogenation of dihydropyrimidinones **88** and dihydropyrimidines **89** via catalytic copper salts and K_2_CO_3_ as base along with TBHP as the terminal oxidant (Scheme 32). The desired compounds **90** and **91** were obtained in excellent yields (Scheme 32) [92].

**Scheme 33:**
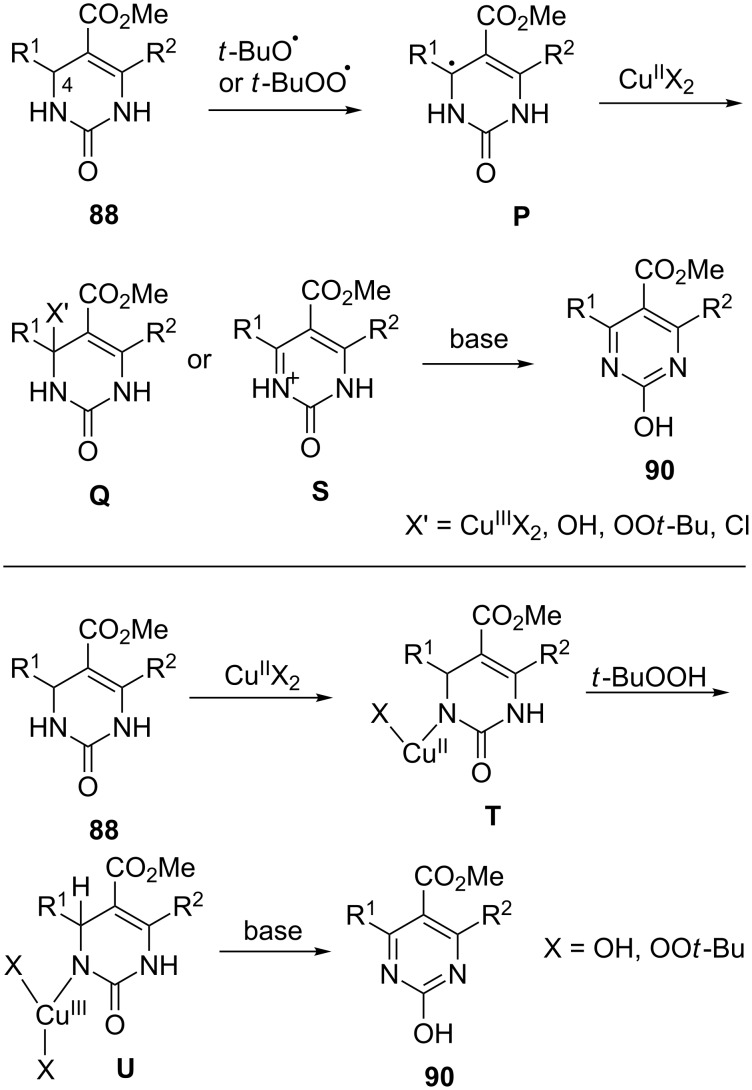
Putative mechanisms (radical and metal-catalyzed) of the transformation.

Two mechanisms were proposed for this conversion. In one the *tert*-butyl peroxy radicals were generated by the interaction of Cu salts with TBHP. This radical abstracts the C4 hydrogen of the dihydropyrimidine to generate **P**, which would then react with Cu(II) to provide species such as **Q** or iminium ion **S**. Either of them would aromatize to generate the desired product **90**. The alternate proposal involved a ligand exchange between Cu(II)X_2_ and dihydropyrimidinone **88** providing compound **T**, which gets oxidized in the presence of TBHP to generate **U** which undergoes further reductive elimination to afford **90** (Scheme 33).

**Scheme 34:**
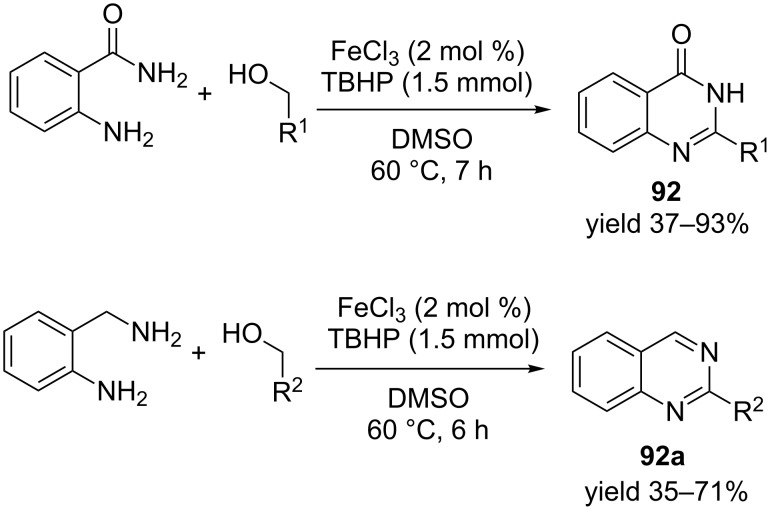
Ferric chloride-catalyzed, TBHP-oxidized synthesis of substituted quinazolinones and arylquinazolines.

A ferric chloride (FeCl_3_)-catalyzed *tert*-butyl hydroperoxide (TBHP)-mediated synthesis of 2-arylquinazolin-4(1*H*)-one **92** was reported by Zhao et al. *o*-Aminobenzamides are reacted with diverse alcohols with 2 mol % of ferric chloride in the presence of *tert*-butyl hydroperoxide (5.5 M in decane, 1.5 mmol) to furnish the final compounds within 7 hours (Scheme 34). This optimized protocol displayed robust functional group tolerance and was further utilised to synthesise aromatic 2-arylquinazoline with excellent yield [93].

**Scheme 35:**
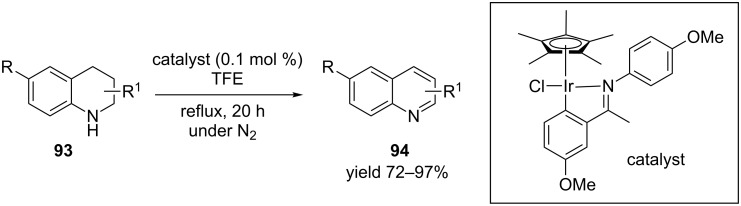
Iridium-catalyzed oxidative dehydrogenation of quinolines.

Recently Xiao et al. [94] has reported an acceptor-free oxidative dehydrogenation with a versatile iridium catalyst (Scheme 35). A variety of tetrahydroquinolines **93** were converted to the aromatic quinoline **94** using the iridium catalyst in 2,2,2-trifluoroethanol (TFE) at higher temperature. As an extension of the optimized reaction conditions tetrahydroquinoxalines were also converted into quinoxalines in excellent yields. The high activity and broad substrate scope of this catalytic system make this protocol very promising for laboratory and industrial applications.

The use of catalytic palladium on carbon in oxidative dehydrogenation was also well explored in the last few decades. In most of these reactions, either an additive or an acceptor is used along with palladium to improve the rate of the reaction. Recently Török et al. [95] reported the synthesis of 1-aryl-β-carboline using montmorillonite K-10 and Pd/C as catalysts.

**Scheme 36:**
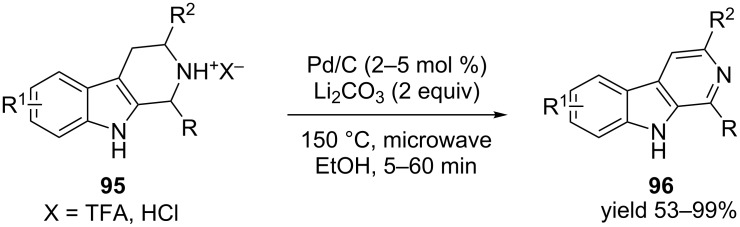
Microwave-assisted synthesis of β-carboline with a catalytic amount of Pd/C in lithium carbonate at high temperature.

The microwave-assisted synthesis of β-carboline **96** from tetrahydro-β-carboline **95** using catalytic Pd/C and lithium carbonate at high temperature is also reported [96]. This high yielding procedure gets completed within a few minutes (Scheme 36). Although the reaction conditions were not well tolerated by few sensitive groups, most of the β-carbolines were successfully prepared utilizing this procedure, including the alkaloid harmine with 99% yield. The final product precipitates at the end of the reaction and hence required minimum purification.

#### Organocatalytic aerobic dehydrogenation

**Scheme 37:**
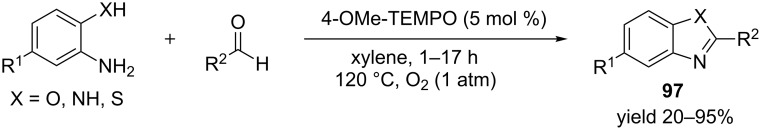
4-Methoxy-TEMPO-catalyzed aerobic oxidative synthesis of 2-substituted benzazoles.

Metal-free organocatalytic aerobic oxidative dehydrogenation of heterocycles is another atom economical and green strategy of accessing various heteroaromatics. Not very many reports are present in the literature to demonstrate this. Hence, the available few render special attention. One such attractive strategy is the 4-methoxy-TEMPO catalyzed aerobic oxidative synthesis of 2-substituted benzoxazoles, benzthiazoles and benzimidazoles **97** [97]. A typical reaction involved 5 mol % of the catalyst heated at 120 °C in oxygen atmosphere with xylene as solvent (Scheme 37). The substrates involved substituted 2-aminobenzophenol, 2-aminobenzothiophenol and *o*-phenyldiamines. A variety of functionalities, e.g., electron-withdrawing or electron-donating, were well tolerated as exemplified by the formation of the desired products **97** in yields of 20–95%.

**Scheme 38:**
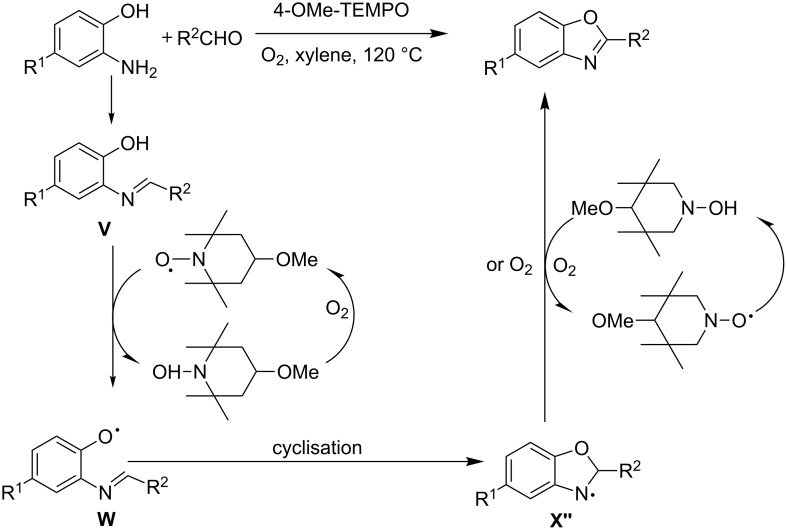
Plausible mechanism of the 4-methoxy-TEMPO-catalyzed transformation.

A plausible mechanism was demonstrated with substituted 2-aminophenol (Scheme 38). Accordingly the reaction is believed to have initiated by the formation of imine intermediate **V**. The 4-methoxy-TEMPO radical interacted with **V** and subsequent H-absorption from the phenol moiety afforded phenoxy radical **W** and 4-methoxy-TEMPOH which gets re-oxidized to the 4-methoxy-TEMPO radical by oxygen. **W** gets stabilized by the imine moiety to form the corresponding amine radical **X′′**. The second hydrogen abstraction between **X′′** and 4-methoxy TEMPO/oxygen facilitates the aromatization to afford the desired compounds (Scheme 38).

**Scheme 39:**
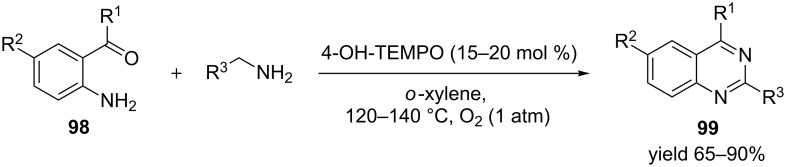
One-pot synthesis of 2-arylquinazolines, catalyzed by 4-hydroxy-TEMPO.

In another example 4-hydroxy-TEMPO was used as a catalyst for the synthesis of 2-arylquinazolines **99** from a one-pot reaction involving arylmethamines with 2-aminophenylketones **98** or aldehydes in the presence of oxygen (Scheme 39) [98]. Diversely substituted 2-arylquinazolines were synthesized in moderate to excellent yields.

### Application of oxidative dehydrogenation in the syntheses of bioactive natural products and drug intermediates

The application of oxidative dehydrogenation in the syntheses of diverse heterocycles makes it a useful strategy for accessing heterocyclic natural products, drug intermediates and active pharmaceutical ingredients. The following section will describe a few of such efforts.

**Scheme 40:**
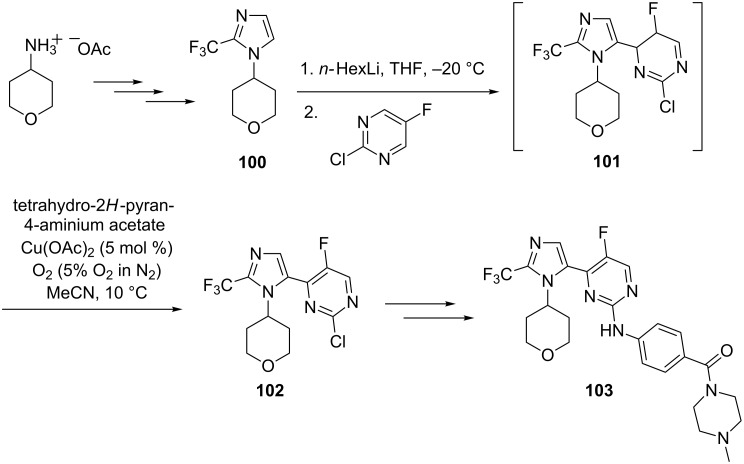
Oxidative dehydrogenation – a key step in the synthesis of AZD8926.

In 2013, Witt et al. reported a scalable process for the synthesis of GSK3B inhibitor AZD8926, **103** [99]. The drug is therapeutically useful for the treatment of CNS disorder viz. Alzheimer’s disease (AD), Schizophrenia and other chronic and acute neurogenerative diseases [99]. This molecule reached the clinical development phase, consequently a safe, robust and economic scale-up process was investigated by the researcher as the original process was incompatible towards scale-up. The investigation indeed provided a better scale-up process, where the key reaction, a Zeigler coupling, of an early trifluoromethylimidazole intermediate **100** with commercially available 2-chloro-5-fluoropyrimidine afforded **101** (a compound with partially saturated pyrimidine framework). An aerobic oxidative dehydrogenation of **101** in the presence of molecular oxygen and with catalytic copper acetate (Cu(OAc)_2_) generated the desired pyrimidine **102** which was converted to the final products in a few more steps. This is one of the noteworthy examples where oxidative dehydrogenation has been utilized for the synthesis of a key intermediate of an active pharmaceutical ingredient (API). This further establishes oxidative dehydrogenation as an impactful strategy in the repertoire of organic synthesis (Scheme 40).

**Scheme 41:**
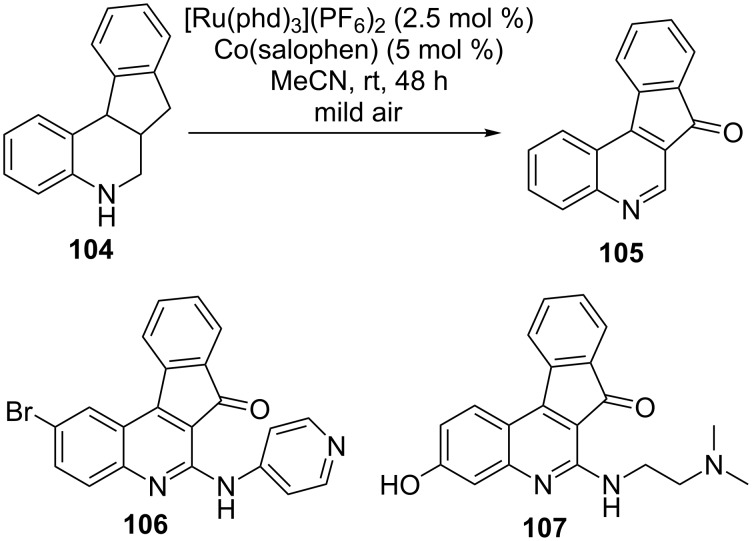
Catalytic oxidative dehydrogenation of tetrahydroquinolines to afford bioactive molecules.

The next example demonstrates the process of oxidative dehydrogenation in the synthesis of bioactive natural products. Accordingly, Stahl et al. utilized a modular catalytic system comprised of C-quinones for the oxidative dehydrogenation of tetrahydroquinolines **104** to an (2,1-*c*)quinoline **105**. This was further transformed to **106**, an antiprotozoal agent, and **107**, a topoisomerase inhibitor, presently in the phase II clinical trial (Scheme 41). The catalytic system comprised of an octahedral [Ru(phd)_3_]^2+^ catalyst along with cobalt-*N*,*N*´-bis(salicylidene)-1,2-phenylenediamine [Co(Salophen)] as a redox co-catalyst. The reaction occurs aerobically under mild conditions (Scheme 41) [100].

**Scheme 42:**
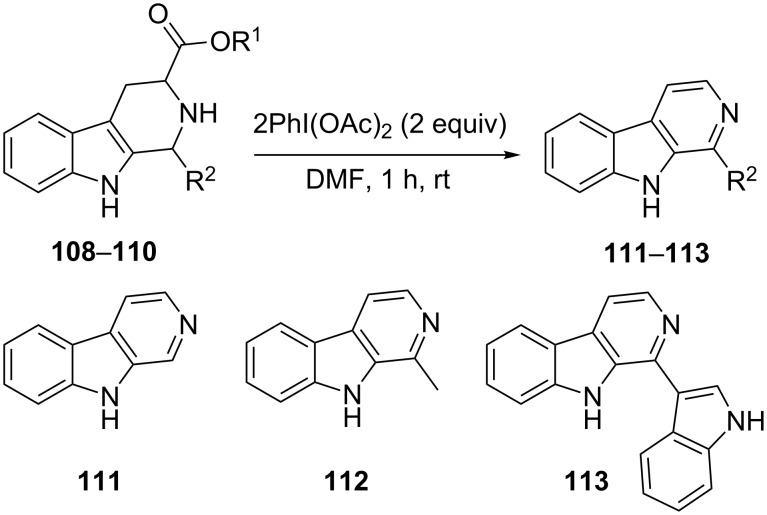
Iodobenzene diacetate-mediated synthesis of β-carboline natural products.

Our final example demonstrated the utility of oxidative dehydrogenation in the synthesis of β-carboline natural products norharmine (**111**), harmane (**112**) and eudistomin U **(113**) [101]. The general synthetic scheme involved the synthesis of the respective tetrahydro-β-carboline precursors of the said natural products by Pictet–Spengler reaction of tryptophan derivatives with appropriate aldehydes. A hypervalent iodine reagent, iodobenzene diacetate was used in stoichiometric quantities to facilitate both oxidative decarboxylation/dehydrogenation of **108**–**110** to afford the desired natural products **111**–**113** (Scheme 42).

## Conclusion

Substantial amount of research has been performed in the last decade demonstrating the efficiency of oxidative dehydrogenation of heterocycles as an effective pathway to access various heteroaromatics. The quantum of publications has increased in this direction where the strategy evolved from stoichiometric → catalytic → organocatalytic. Diverse oxidants such as NBS, DDQ, KMnO_4_, IBX, DIB and last but not least molecular oxygen have been used as an oxidant for this purpose. Recently bioinspired catalysts have also been developed. As discussed in this review, the processes afforded the desired compounds in moderate to excellent yields and are robust enough to be applied in the generation of natural products and synthetic drug intermediates. This review will serve as a compilation of the published methods where oxidative dehydrogenation is harnessed to generate various heteroarenes and we sincerely hope that this will arouse interest among medicinal and synthetic organic chemists to utilize this methodology to access them.
